# Hyper-acute effects of sub-concussive soccer headers on brain function and hemodynamics

**DOI:** 10.3389/fnhum.2023.1191284

**Published:** 2023-09-14

**Authors:** Carissa Grijalva, Dallin Hale, Lyndia Wu, Nima Toosizadeh, Kaveh Laksari

**Affiliations:** ^1^Department of Biomedical Engineering, University of Arizona, Tucson, AZ, United States; ^2^Department of Physiology, University of Arizona, Tucson, AZ, United States; ^3^Department of Mechanical Engineering, University of British Columbia, Vancouver, BC, Canada; ^4^Arizona Center for Aging, Department of Medicine, University of Arizona, Tucson, AZ, United States; ^5^Department of Aerospace and Mechanical Engineering, University of Arizona, Tucson, AZ, United States

**Keywords:** subconcussive, hyperacute, fNIRS (functional near infrared spectroscopy), soccer, TCD (transcranial doppler sonography), dual task

## Abstract

**Introduction:**

Sub-concussive head impacts in soccer are drawing increasing research attention regarding their acute and long-term effects as players may experience thousands of headers in a single season. During these impacts, the head experiences rapid acceleration similar to what occurs during a concussion, but without the clinical implications. The physical mechanism and response to repetitive impacts are not completely understood. The objective of this work was to examine the immediate functional outcomes of sub-concussive level impacts from soccer heading in a natural, non-laboratory environment.

**Methods:**

Twenty university level soccer athletes were instrumented with sensor-mounted bite bars to record impacts from 10 consecutive soccer headers. Pre- and post-header measurements were collected to determine hyper-acute changes, i.e., within minutes after exposure. This included measuring blood flow velocity using transcranial Doppler (TCD) ultrasound, oxyhemoglobin concentration using functional near infrared spectroscopy imaging (fNIRS), and upper extremity dual-task (UEF) neurocognitive testing.

**Results:**

On average, the athletes experienced 30.7 ± 8.9 g peak linear acceleration and 7.2 ± 3.1 rad/s peak angular velocity, respectively. Results from fNIRS measurements showed an increase in the brain oxygenation for the left prefrontal cortex (PC) (*p* = 0.002), and the left motor cortex (MC) (*p* = 0.007) following the soccer headers. Additional analysis of the fNIRS time series demonstrates increased sample entropy of the signal after the headers in the right PC (*p* = 0.02), right MC (*p* = 0.004), and left MC (*p* = 0.04).

**Discussion:**

These combined results reveal some variations in brain oxygenation immediately detected after repetitive headers. Significant changes in balance and neurocognitive function were not observed in this study, indicating a mild level of head impacts. This is the first study to observe hemodynamic changes immediately after sub-concussive impacts using non-invasive portable imaging technology. In combination with head kinematic measurements, this information can give new insights and a framework for immediate monitoring of sub-concussive impacts on the head.

## 1. Introduction

There are approximately 1.9 million sports-related concussions (SRC) diagnosed annually in the U.S. youth population alone, and many incidences have historically been underreported (Centers for Disease Control and Prevention, 2019). The diagnosis of concussion itself relies on subjective symptoms, as currently there is no consensus with conventional MRI/CT (McCrory et al., 2017). Researchers have correlated head kinematics with brain injury by recording real time linear acceleration and angular velocity on the field using 6 degrees-of-freedom wearable sensor technology embedded into mouth guards (Hernandez et al., 2015; Wu L. C. et al., 2018; Laksari et al., 2022). Concussive levels of impact exposure typically range between 50 and 100 g of head acceleration (Laksari et al., 2018, 2019), whereas sub-concussive impacts range between 10 and 30 g (Wu et al., 2016; Nguyen et al., 2019). However, there is less focus on sub-concussive impacts in sports participation and defining the effect of these repeated impacts on brain’s function. Sub-concussive impacts are described as a cranial impact resulting in rapid head acceleration similar to a concussion, but with less impact force and not resulting in clinical diagnosis of concussion (O'Keeffe et al., 2020). The repetitive exposure to sub-concussive head impacts has demonstrated differing brain activity, cognitive impairments, as well as long term neurological deficits for some individuals (Montenigro et al., 2017). These methods have demonstrated some brain changes (Di Virgilio et al., 2016, 2019), however, hyper-acute (within minutes after impacts) physiological response in terms of cerebral hemodynamics has not been studied rigorously.

Soccer athletes frequently sustain sub-concussive head impacts, since they intentionally use the unprotected head to direct the ball, known as “soccer heading.” Players may experience an average of 6–12 heading incidents per game (Spiotta et al., 2012) leading up to thousands of sub-concussive head impacts in a single season (Bailes et al., 2013), with ball velocities that may reach up to 25 m/s (Kirkendall and Garrett, 2001). However, there is limited data associating soccer headers with neurological function of the brain immediately after impact.

Cerebrovascular changes have been reported as indirect results of SRC, which may become chronic if subjected to repetitive head impacts (Bailes et al., 2013). This secondary injury pathology may include disruption of the blood brain barrier, impaired cerebral autoregulation, or changes in cerebral blood flow velocities (CBFV) (Giza and Hovda, 2014; Churchill et al., 2017). This has been demonstrated via functional MRI (fMRI) studies of brain oxygenation changes in sports athletes from pre- to post-season, resulting in both hyper-activation (Talavage et al., 2014) and hypo-activation (Keightley et al., 2014) within the frontal lobe during task-related functions. There is a present link between acute and long-term alterations in cerebral hemodynamics following concussions, however, these studies measure changes within days, weeks, or months post-injury (Smits et al., 2009; Hammeke et al., 2013).

Currently, there is a lack of understanding on how sub-concussive head impacts affect cerebral perfusion at the hyper-acute level, i.e., immediately after impact. The difficulty with common imaging modalities such as MRI/fMRI, is the time for travel, setup, and scanning, which makes measuring hyper-acute changes in the brain challenging. There are currently other advanced non-invasive neuroimaging devices with a unique portable capability which include the transcranial Doppler (TCD) ultrasound and functional near infrared spectroscopy (fNIRS). These methods allow for real-time measurements of real-world head impact effects on cerebral hemodynamics (Bathala et al., 2013; Bishop and Neary, 2018). Thus, results can be obtained on-field in a non-conventional setting to observe the most immediate neurophysiological effects from impacts.

TCD can measure blood flow velocity from various branches of the Circle of Willis (Sarabian et al., 2022) via transducers sending 2–16 MHz wave pulses, which are reflected by moving red blood cells (Fw, 2006). The middle cerebral artery (MCA) is one of the larger arteries in the brain supplying blood to the frontal lobe. This artery can be accessed via the trans-temporal window and has been shown to change following soccer heading (Scale et al., 1996). There are limited TCD measurements from athletes following concussion which have shown changes in the MCA blood flow velocity (Clausen et al., 2016; Smirl et al., 2020).

The fNIRS imaging modality emits near infrared light (~650-900 nm) and collects signals based on the optical absorption of the oxygenated (O2Hb) and deoxygenated (HHb) forms of hemoglobin in the tissue (Clancy et al., 2014). The modified Beer–Lambert Law is used to convert the quantity of light absorbed to change in concentration of hemoglobin (Delpy et al., 1988). The use of fNIRS in the context of concussion has been limited and the method of data processing is still being researched (Herold et al., 2018). One study had used fNIRS to distinguish between traumatic brain injury (TBI) subjects and healthy controls demonstrating diminished brain oxygenation patterns for TBI patients (Karamzadeh et al., 2016). fNIRS has shown potential applications for distinguishing between TBI and healthy subjects, especially in oxygenation and autoregulation monitoring (Roldan and Kyriacou, 2021), making it suitable to measure immediate changes in brain function.

Standardized assessments have demonstrated effective use in measuring parameters from individuals diagnosed with concussions, since they commonly suffer from deficits in sensorimotor function or balance (Wood et al., 2019). There is a validated test for predicting neurocognitive and motor performance called the upper extremity function (UEF) dual-task test (Toosizadeh et al., 2019). The usefulness of UEF dual-task actions to identify cognitive status has been previously demonstrated in older adults (Toosizadeh et al., 2016), and also used to assess various neurological related diseases including frailty (Toosizadeh et al., 2017), patients with mild cognitive impairment (Grijalva et al., 2021), and Alzheimer’s Disease (Toosizadeh et al., 2019). We expected this measure to be sensitive enough to detect motor and cognitive changes at the sub-concussive level. This test uses wearable sensor technology to measure cognition and physical function status by monitoring simultaneous performance of repetitive arm movement and counting, known as dual-tasking, which could be affected following sub-concussive head impacts (Toosizadeh et al., 2017).

The objective of this study was to apply novel mobile sensing tools including head impact sensors and neuroimaging devices with neurological assessments to examine the hyper-acute, i.e., within minutes, effects of sub-concussive soccer heading on neurological function and brain hemodynamics. Based on previous concussion and soccer studies (Kontos et al., 2014; Caccese et al., 2019), we hypothesized increased changes in blood flow velocity profiles after experiencing head impacts, as well as significant changes in brain oxygenation, cognitive abilities, and balance parameters, which would be correlated to the head kinematics experienced. Additionally, based on previous research of higher rates of concussion in the female group (Marar et al., 2012), we hypothesized any changes observed would also be greater for the females compared to males.

## 2. Materials and methods

### 2.1. Participants

The study protocol was reviewed and approved by the Human Subjects Institutional Review Board at the University of Arizona (1905625800). Twenty healthy university-level soccer athletes (*n* = 10 female, *n* = 10 male, age > 18, all right-handed) with previous soccer experience were recruited to participate in this study (Table 1). Exclusion criteria comprised diagnosed diseases associated with motor performance deficits or known history of brain injury or concussion within the last 6 months. An additional 5 subjects were analyzed as a control group with no head contact. Written informed consent was obtained from all participants before participation and all research was performed in accordance with guidelines from the Declaration of Helsinki (World Medical Association, 2013).

**Table 1:** Demographic parameters.

Characteristic	Male	Female	*p* value
*n*	10	10	
Age (years)	21.6 ± 2.9	20.5 ± 2.6	0.42
Height (cm)	175.26 ± 6.7	165.8 ± 7.1	0.01
Weight (lbs)	176.2 ± 32.8	132.7 ± 9.6	0.01
Years of experience	12.5 ± 3.3	14.1 ± 2.2	0.22

### 2.2. Participant instrumentation

**Figure 1:**
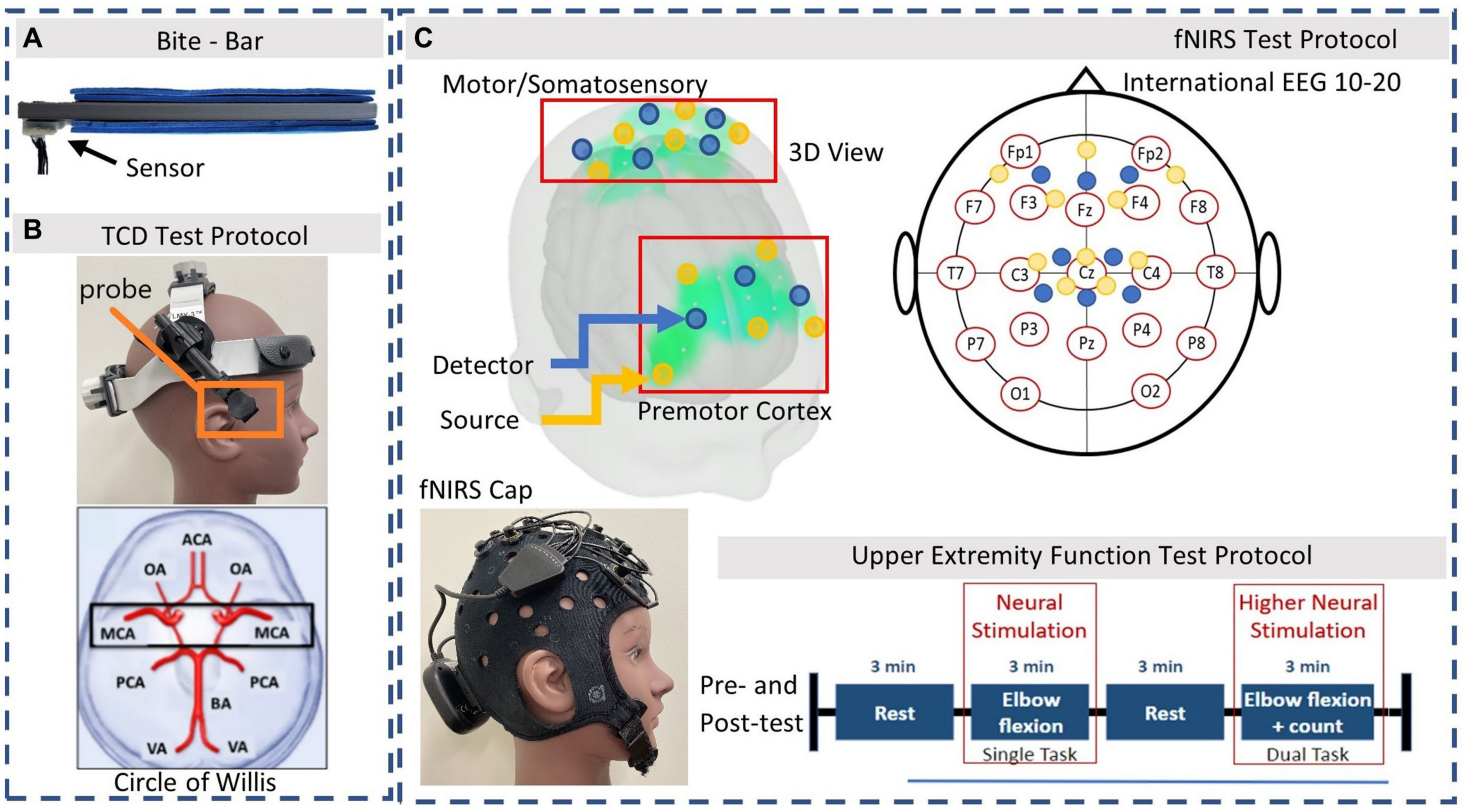
Instrumentation. **(A)** 3D printed bite-bar with embedded sensor, worn during soccer headers. **(B)** TCD headset and Axial slice view of MCA. **(C)** fNIRS optodes arrangement based on the international EEG 10–20 system. Source and detector pairs are demonstrated in the 3D view: Yellow = source, Blue = detector. The Upper Extremity test protocol is performed during the fNIRS measurements system.

Participants were equipped with a 3D-printed rigid bite-bar instrumented with a 6 degree-of-freedom inertial measurement unit (IMU) (ICM-20649) while performing soccer headers (Figure 1A). The sensor containing a tri-axial accelerometer and gyroscope (sample frequency = 1,000 Hz) was rigidly attached at the end of the bite-bar with glue extending outside the mouth and controlled *via* IMU Arduino software.

During the laboratory assessments participants were equipped with a TCD headset (Rimed, Digi-Lite, New York, USA) to measure both the left and right MCA velocity with 2 MHz transducers fixed in place (Figure 1B). The TCD M1 MCA depths were assigned as 65–45 mm distance from the transducer accessed *via* the trans-temporal window and confirmed with direction of blood flow according to previous studies (Alexandrov et al., 2002). The same trained personnel recorded all TCD signals. Participants were then separately equipped with the fNIRS (Brite 24 Artinis Medical Systems, Netherlands) device to measure brain oxygenation at a sample frequency of 50 Hz *via* multi-wavelength LEDs situated on a soft neoprene head-cap containing 8 receivers and 10 transmitters 30 mm apart, allowing for approximately 15 mm penetration depth. The probe positions of the fNIRS detection device covered the area linking Fp1, F3, F7, and Fp2, F4, F8, corresponding to the left and right prefrontal cortex respectively, as well as C3, Cz, C4, corresponding to the primary motor cortex and somatosensory cortex, according to the international EEG 10–20 system (Figure 1C; Okamoto et al., 2004). Changes in light attenuation were measured at two wavelengths (763 and 842 nm) and concentration change was calculated using the modified Beer– Lambert law within the Oxysoft software. The differential path length factor (DPF) was calculated for each individual in relation to their age which accounts for the increase in optical pathlength due to scattering in the tissue (Delpy et al., 1988). Data collected included oxyhemoglobin (O2Hb) and deoxyhemoglobin (HHb) concentrations *via* OxySoft software. To ensure good signal from the fNIRS optodes, the cap was tightly fitted, properly secured, and any hair was moved aside, with signal quality verified in real time.

During fNIRS measurements, participants wore two wireless accelerometer and gyroscope sensors (sample frequency = 100 Hz, BioSensics LLC, Brookline, MA, USA) on the bicep and wrist of the dominant arm using a Velcro band, to perform the motor function UEF test. This test was easily performed and post-processed in minutes. These sensors were also used to measure balance parameters when placed on the waist and ankle as the participant stood still for 30 s. Lastly, participants were also equipped with a wireless electrocardiogram (ECG) sensor (360° eMotion Faros, Mega Electronics, Kuopio, Finland) attached across the lower torso to measure heart rate (HR) at a sampling frequency of 1,000 Hz, during fNIRS recordings and during soccer headers. HR was not expected to increase due to minimal motion or exercise involved in the controlled heading experiment. Any changes observed were expected to occur from head impact and not increased stress or HR.

### 2.3. Experimental protocol

Participants were evaluated using a design that involved a pre-test, head impact exposure (soccer heading), and a post-test. Measurements were completed within 20 min following 10 consecutive soccer headers to determine hyper-acute responses. These measurements consisted of the following: (1) TCD recordings (~2 min), (2) fNIRS with UEF test and ECG (~15 min), (3) balance measurements (~1 min) in this order, while continuously measuring HR. Participants were seated during TCD and fNIRS measurements. First, resting velocities of the MCA were recorded for approximately 2 min using the TCD headset. Following this, participants wore the fNIRS cap and the wireless arm sensors, and performed the UEF dual-task motor and cognitive test (Toosizadeh et al., 2016). This consisted of 2 task periods (single task and dual task) with rest periods in between, with each period lasting 3 min (Figure 1C). The single-task (ST) movement consisted of bending and straightening the dominant arm as consistently as possible. The dual-task (DT) movement consisted of the same motion while also counting backwards by three, from a given number. Participants followed instructions from a visual presentation in an enclosed room near the outdoor field, to minimize distraction. A practice trial was provided before fNIRS data acquisition to minimize learning effects. Trained personnel were able to place the TCD headset or fNIRS cap on the participant and optimize the signal within 2 min for each device before and after the head impact exposure. Following this, participants performed a balance test where they stood still in place for 30 s with their eyes open, and then repeated this with their eyes closed.

Participants performed 10 consecutive soccer headers outdoors, within a period of 7–10 min to replicate typical heading during practice (Spiotta et al., 2012; Smirl et al., 2020). Soccer balls were delivered from a ball launcher device (Sports Tutor Inc. Burbank, CA), which was set at a launch velocity of approximately 15 m/s for mild head impacts. The ball was shot an angle of approximately 35 degrees with the participant standing 24 m away. Size 5 soccer balls (GoSports, P&P Imports, LLC) were used and inflated to the manufacturer’s guidelines between 8 and 9 psi and confirmed with a pressure gauge. Each participant was instructed to contact the ball on the forehead without jumping or moving to either side. If there was no contact with the ball, another soccer ball was launched within the 30 s window and repeated until a successful header occurred. In the case of the 5 control participants, the same pre- and post-measurements were taken from the fNIRS device, however, during the “soccer” period, participants instead stood outside in the same location and only mimicked the movement of the soccer header 10 times over the course of 7–10 min.

### 2.4. Data analysis

All kinematic sensor data from the bite bar were filtered with a second-order Butterworth low pass filter with 300 Hz and 184 Hz cutoff frequencies for the accelerometer and gyroscope, respectively. Angular acceleration data were computed numerically by differentiating gyroscope data using a five-point differential formula in MATLAB (Miller et al., 2018). The bite-bar sensor kinematic data were converted to the head’s coordinate system at the head center of gravity (CG) according to the previously used rigid body transformation equation (Miller et al., 2019).

Images from the TCD ultrasound were exported and peaks were extracted over the course of 2 min, approximately 10 peaks over the duration of 8-s time increments which was done in a graph digitizing software. The step interpolation algorithm was used to obtain the contour trace of the velocity profile as data points with the known axis limits. The right and left MCA blood flow velocity data were averaged together for each measurement.

The fNIRS signal was down-sampled to 5 Hz when exported from Oxysoft software (Artinis Medical Systems, Netherlands). All data was read into MATLAB Fieldtrip Toolbox for analysis (Oostenveld et al., 2011). Channels were visually inspected on the screen during data collection and during analysis, for the presence of large motion artifacts, and to certify that heartbeat oscillations were occurring indicating a good optical coupling between the optodes and the scalp (Pinti et al., 2019). Bad channels due to poor scalp coupling were removed within the Fieldtrip toolbox. This was done by using the optical density traces which should have a heart beat that is positively correlated, if not, the optode was excluded (Pollonini et al., 2014). Motion correction is performed using a built-in function called ft_artifact_zvalue in the Fieldtrip toolbox in MATLAB. This scans the data segments of interest for artifacts due to motion, by means of thresholding the z-scored values of signals that have been preprocessed using heuristic properties to increase the sensitivity to detect certain artifacts (Oostenveld et al., 2011). Channels that contain a signal in which the peak-to-peak range within the trial exceeds this threshold are selected. The z-score is applied to make the threshold independent of physical units in the data, and the output is an Nx2 matrix with the first column specifying the begin samples of the artifact period, and the second column specifying the end samples of the artifact period (Oostenveld et al., 2011). Then these selected matrices are removed by using the built in function called ft_rejectartifact which removes data segments containing the artifacts. The data was then converted into concentration values using the modified Beer Lambert Law (Delpy et al., 1988). Cardiac (1–2 Hz) and respiration (0.2–0.4 Hz) interference in the signal were removed using a band-pass filter with cut-off frequencies of 0.001–0.1 Hz (Li et al., 2021), while still extracting important physiological signal due to the task (stimulation frequency = 1/180 s = 0.005 Hz). Next, Principal Component Analysis (PCA) was done to remove global interference. Each component had an associated eigenvalue, and the component with the largest value was likely due to the most interference from surface tissue (Mäki et al., 2010). This was the first principal component and the time course was then removed in later analysis. A preferred method of removing additional physiological factors in fNIRS experiments is using the General Linear Model (GLM) (Cohen-Adad et al., 2007; Ye et al., 2009), which consists of regressing fNIRS data with a linear combination of regressors and an error term. GLM measures the temporal variational pattern of signals rather than their absolute magnitude and incorporate regressors such as scalp blood flow, into the statistical framework (Ye et al., 2009; Tachtsidis and Scholkmann, 2016). The GLM regression analysis was applied to all channels using the MATLAB FieldTrip Toolbox (Oostenveld et al., 2011). Briefly, we read in “events,” which are represented as triggers, indicating the samples in the data in which the task started or stopped. We use these to make some additional continuously represented channels that represent the onset, offset, and the motion. We include two channels for a constant offset, and for a slope. These are used to remove the baseline and a constant drift in the signal over time. We perform GLM analysis where each channel is represented as a pixel in the statistical parametric map (SPM) which is done to statistically analyze and compare groups of images to highlight neurological differences (Ye et al., 2009). To create a regressor of interest, the hemodynamic response is predicted by convolving the canonical hemodynamic response function (HRF) and its temporal and dispersion derivatives included, with a single boxcar function regressor that represents the task segments (Schroeter et al., 2004; Koh et al., 2007; Lindquist et al., 2009). A boxcar function was used due to the required sustained stimulation of performing motor and cognitive tasks in our study (Schroeter et al., 2004; Wu Z. et al., 2018). The first and second derivatives control the timing of the peak response as well as the width. The design matrix consisted of one regressor of interest (single or dual task) convolved with the HRF, and added nuisance regressors including the signal drift and the time course of the first principal component to be removed. After GLM analysis of the channels, results were grouped together based on functional regions, i.e., prefrontal, motor/sensorimotor for concentration changes in O2Hb. Results were plotted and peak concentrations during the task regions were assessed.

Analysis of the fNIRS signals included concentration changes in O2Hb, and sample entropy analysis. Sample entropy calculations were performed on pre- and post-header measurement data as a metric for signal complexity using a custom MATLAB code. It is calculated as SampleEntropy (m,r,N) = −ln(A(m)/B(m)). This is defined as the negative logarithm of the conditional probability that A(m) – two sequences are similar for m points (possibles) –, and B(m) – two sequences are similar for m + 1 points (matches) –, that match pointwise within a tolerance r and small number of points N (Delgado-Bonal and Marshak, 2019). For example, an ideal periodic time series is more predictable and would have zero sample entropy; whereas, large sample entropy values would represent less repeatability and higher variability (Lamoth et al., 2011). Lastly, to assess changes in an individual’s performance from a single to a dual-task, dual-task “cost” was calculated as the percentage of change within the conditions. This was obtained by normalizing the DT performance for each participant using the ST baseline to assess compromised motor/cognitive parameters ([DT – ST]/ST).

Angular velocity and anthropometric data of the UEF elbow flexion test, i.e., participants’ stature and body mass, were used to define the UEF neurocognitive score for each participant. Lastly, the ECG signal was analyzed using an in-home peak detection MATLAB algorithm for beat-to-beat HR. Briefly, the data from the HR sensor was read into the MATLAB app and separated into regions of ST, DT, Rest, and the soccer measurement was analyzed separately. These regions were marked by pushing the sensor button to mark the event during the experiments. ECG data was analyzed for 20 s of baseline, 180 s of UEF (for the fNIRS measurements ST and DT), and 30 s of recovery. RR intervals (successive R peaks of the QRS signal) were computed using the Pan-Tompkins algorithm (Pan and Tompkins, 1985). Next, the automated peak detection process was manually inspected. Output parameters extracted were baseline HR (the HR before the task began), as well as change in HR during the UEF task (Toosizadeh et al., 2022). The same analysis was done for signals recorded during the soccer headers. We measured changes in HR during the fNIRS task and rest blocks as well as during soccer heading for comparison.

All data was assessed for normal distribution using the Shapiro–Wilk test (Shapiro et al., 1968). Statistical paired t-tests were used for normally distributed groups and Wilcoxon non-parametric tests were used for non-normally distributed groups to determine significant difference between the pre- and post-header measurements across participants. Statistical significance was set at *p* ≤ 0.05 for all analyses. Cohen’s d was applied to determine effect size with the numerator being the difference between the means of the two observations, and the denominator being a pooled standard deviation (Lakens, 2013).

## 3. Results

### 3.1. Kinematics

**Figure 2:**
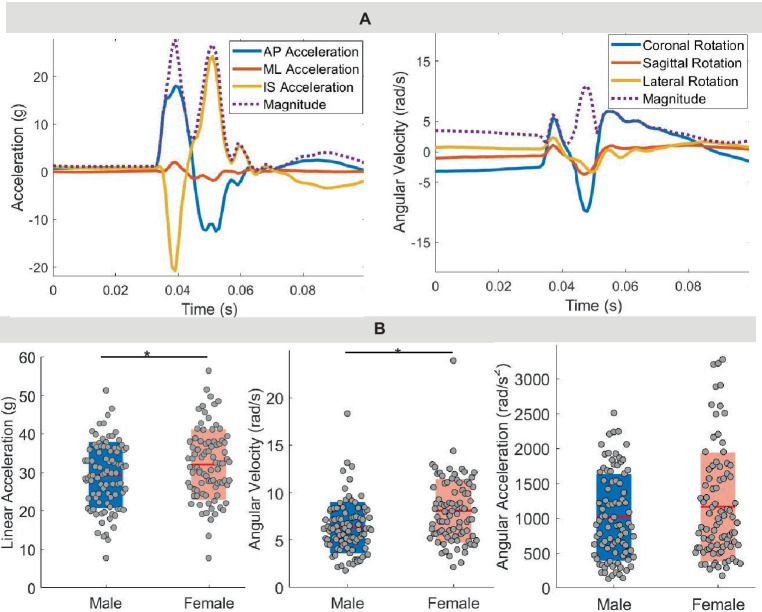
Kinematics. **(A)** Time trace for linear acceleration and rotational velocity from performing one soccer header. AP, anterior–posterior; ML, medial-lateral; IS, inferior–superior. **(B)** Peak linear acceleration, peak rotational velocity, and peak rotational acceleration across all participants.

During the heading protocol, 172 successful headers were recorded by 18 participants with an average peak head linear acceleration of 30.6 ± 8.9 g, peak angular velocity of 7.2 ± 3.1 rad/s, and peak angular acceleration of 1,084 ± 700 rad/s^2^ (Figure 2; Table 2). The data from the mouth guard were corrupted for two participants. Female participants experienced a significantly higher linear acceleration (*p* = 0.01) and angular velocity (*p* < 0.0001) compared with males, but we observed no significant differences in angular acceleration (*p* = 0.17). According to sensor orientation, these forehead impacts typically resulted in higher linear accelerations in both vertical and forward directions while rotational motion was predominantly in the sagittal and coronal directions.

**Table 2:** Kinematic and TCD results.

Parameters	All	Male	Female	*p* value	Effect size
Linear acceleration (g)	30.7 ± 8.9	29.3 ± 8.5	32.1 ± 9.01	*0.01	0.32
Angular velocity (rad/s)	7.2 ± 3.1	6.3 ± 2.7	8.1 ± 3.3	*<0.0001	0.55
Angular acceleration (rad/s^2^)	1,084 ± 700	1,012 ± 614	1,160 ± 776	0.17	0.2
MCA blood flow velocity (cm/s)					
Pre-header	83.2 ± 10.2	80.2 ± 7.9	86.5 ± 11.9	0.09	0.58
Post-header	83.7 ± 10.8	79.8 ± 8.5	88.1 ± 11.8	*0.04	0.76
*p* value (pre vs. post)	0.26	0.31	0.17		
Effect size (pre vs. post)	0.05				
Sample entropy MCA					
Pre-header	0.22 ± 0.03	0.21 ± 0.02	0.24 ± 0.01	*0.002	1.7
Post-header	0.21 ± 0.02	0.2 ± 0.02	0.22 ± 0.02	0.07	0.93
*p* value (pre vs. post)	0.1	0.44	0.07		
Effect size (pre vs. post)	0.4				
Pulsatility index MCA					
Pre-header	0.94 ± 0.09	0.9 ± 0.06	0.98 ± 0.09	*0.02	0.97
Post-header	0.93 ± 0.1	0.89 ± 0.08	0.97 ± 0.09	*0.02	0.87
*p* value (pre vs. post)	0.44	0.39	0.37		
Effect size (pre vs. post)	0.11				

MCA, middle cerebral artery. Bold values with an asterisk indicate significance *p* < 0.05.

### 3.2. Pre- and post-header TCD changes

**Figure 3:**
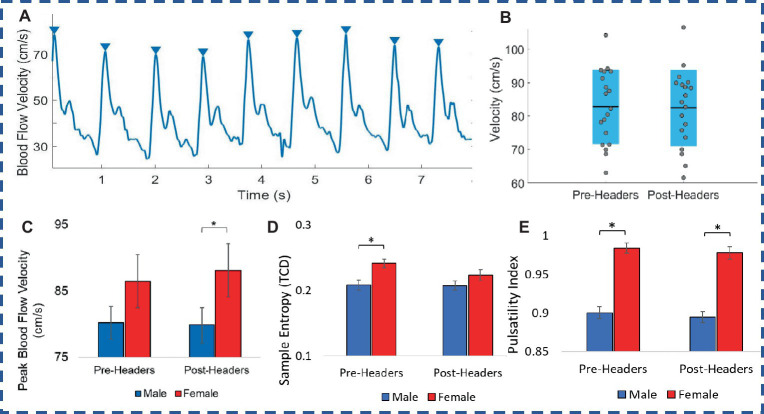
Transcranial doppler. **(A)** Ultrasound recording of blood flow velocity (BFV). **(B)** Male and female comparison of peak BFV. **(C)** Peak BFV levels. **(D)** Sample entropy of BFV. **(E)** Pulsatility index of TCD signal. Error bars represent standard error.

When comparing pre-and post-header measurements from the TCD ultrasound, there were significant changes in MCA velocity in some participants in terms of peak velocities recorded over the 2 min, however there was not a significant change in peak values across all 19 participants (Figure 3; Table 2). The MCA was not able to be located for one participant. Additionally, there was a significant difference between female and male participants in the post-header measurements (*p* = 0.04; Figure 3C; Table 2). Sample entropy analysis and pulsatility index (PI) calculated from the TCD signal also showed some significant changes at the individual level, but this change was not observed on average across participants. However, male and female differences were seen in both the pre- and post-measurements for PI (*p* = 0.02) (Table 2). We also observed a small correlation of participant kinematics and change in blood flow velocity (see Supplementary information).

### 3.3. Pre- and post-header fNIRS changes

**Figure 4:**
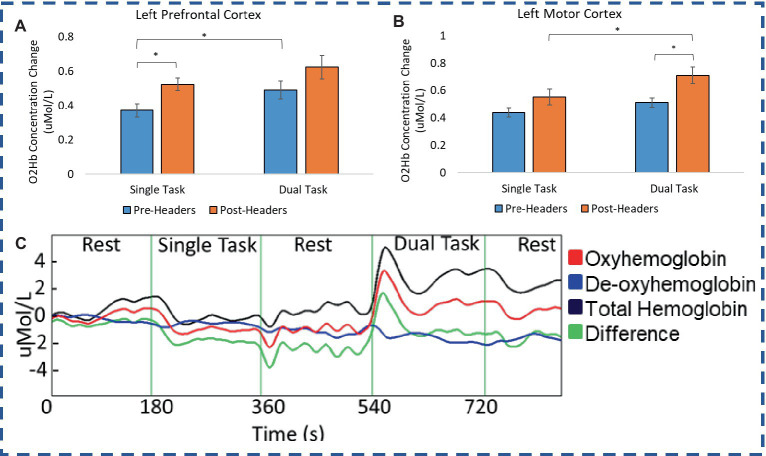
fNIRS concentration. O2Hb concentration changes for **(A)** Premotor Cortex and **(B)** Motor Cortex during the single and dual task UEF test. **(C)** Example time trace of concentration change.

**Figure 5:**
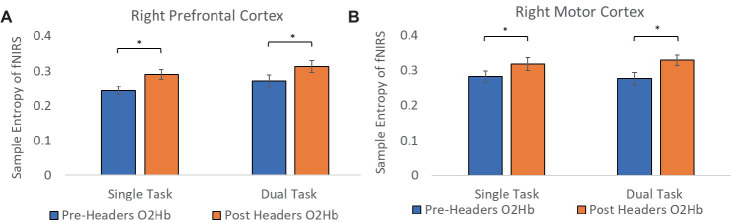
Sample entropy of fNIRS. Sample entropy of oxyhemoglobin concentration pre-post measurements in the **(A)** prefrontal cortex and the **(B)** motor cortex. Error bars represent the standard error.

**Figure 6:**
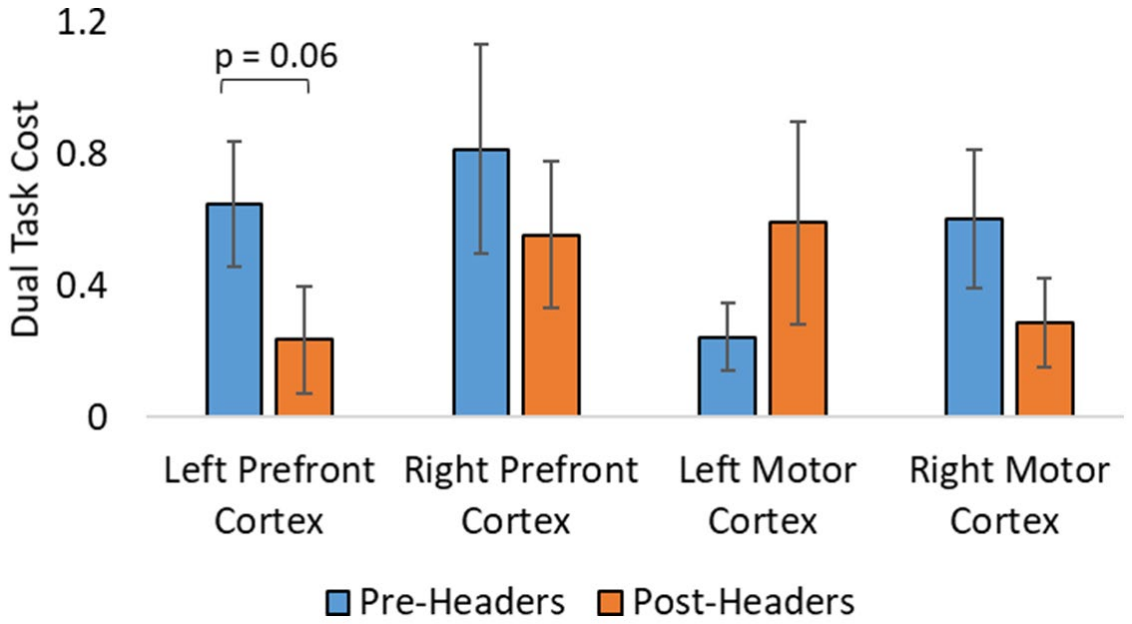
Additional analysis from fNIRS. Dual task cost analysis of fNIRS concentration changes. Error bars represent the standard error.

A total of 19 participants fNIRS data were included in the analysis because one subject did not have sufficient skin-coupling with the sensors. There was an overall increasing trend of O2Hb concentration during the post-header measurements compared with the pre-header measurements following GLM regression. This was significant in the left prefrontal cortex during the ST period (*p* = 0.002) (Table 3; Figure 4). This was also significant in the left motor cortex during the DT period (*p* = 0.007). The right cortex did not show any significance in terms of O2Hb concentration changes. The results of this study report significantly increased sample entropy in post-header measurements of oxyhemoglobin concentration in the right prefrontal cortex for the ST period (20.8%, *p* = 0.007), and the DT period (17.3%, *p* = 0.02), and in the right motor cortex for the ST period (19.3%, *p* = 0.04), and the DT period (17.4%, *p* = 0.004; Figure 5). The left motor cortex ST period was significantly different (16.5%, *p* = 0.04). Additionally, we did not find any significant differences in terms of O2Hb concentration changes for the control participant group in single or dual task (see Supplementary material). Lastly, DT cost function analysis results demonstrate mostly decreased values in the post-header measurements but was not significant for the left prefrontal cortex region (*p* = 0.06; Figure 6).

**Table 3:** fNIRS concentration changes.

	Prefrontal/Premotor cortex				Motor/Somatosensory cortex			
Parameters	Pre-headers	Post-headers	*p* value	Effect size	Pre-headers	Post-headers	*p* value	Effect size
*Single-task*								
Left O2Hb	0.37 ± 0.17	0.52 ± 0.16	*0.002	0.9	0.44 ± 0.14	0.55 ± 0.25	0.05	0.54
Right O2Hb	0.42 ± 0.19	0.45 ± 0.22	0.31	0.15	0.41 ± 0.14	0.48 ± 0.22	0.11	0.38
*Dual-task*								
Left O2Hb	0.49 ± 0.2	0.62 ± 0.29	0.07	0.52	0.51 ± 0.13	0.71 ± 0.26	*0.007	0.97
Right O2Hb	0.55 ± 0.16	0.58 ± 0.28	0.35	0.13	0.52 ± 0.16	0.54 ± 0.17	0.45	0.12

Bolded values represent significance at the level of *p* ≤ 0.05. O2Hb, oxyhemoglobin.

### 3.4. Task-based fNIRS changes

In many cases the more challenging DT period had significantly greater peak O2Hb concentrations (Figure 4). DT concentrations were significantly different compared with the ST period in the left prefrontal cortex (pre-headers *p* = 0.03), and the right prefrontal cortex (pre-headers *p* = 0.02). DT concentrations were also significantly different compared with the ST period in the left motor cortex (post-headers *p* = 0.02), and the right motor cortex (pre-headers *p* = 0.01).

### 3.5. Balance, function, and heart rate

Balance parameters were compared between pre- and post-header measurements but showed no significant differences among the athletes (*p* = 0.46). There were also no significant differences present in the motor and cognition UEF DT performance score when comparing pre- and post-header measurements. Lastly, there was a similar pattern seen in terms of HR. Across the athletes, HR showed significant differences between the rest and task periods before and after a bout of soccer heading (*p* = 0.04). However, HR during soccer heading was comparable to the DT period as there were no significant differences (*p* = 0.19) (see Supplementary material). In addition, the HR range was calculated post-headers and correlated with fNIRS results using Pearson correlation. There was no significant correlation of the HR range with changes in fNIRS values for either the heading and the control group (*p* > 0.09) (see Supplementary material).

## 4. Discussion

The present study explored how hemodynamics are affected after a bout of controlled soccer heading through non-invasive, portable, real-time ultrasound and near infrared spectroscopy measurements of blood flow velocity and oxygenation. We observed significant changes in oxyhemoglobin concentrations *via* fNIRS measures immediately after impacts. Other measures of the fNIRS signal such as sample entropy and DT total cost, also support the regional brain oxygenation changes occurring immediately after headers. This information in combination with kinematic data, can provide more insight to sub-concussive responses in brain function and physiology at the hyper-acute stage.

To our knowledge, this is the first study to use the portable, real-time DT-based fNIRS to explore the immediate effects of sub-concussive level impacts on brain oxygenation changes. Overall, we found increases of O2Hb concentrations for each region of the brain, however, the significant results occur in the left prefrontal and motor cortex. All of the athletes were right-handed, and it was interesting to see the main brain oxygenation patterns that we observe were contralateral to the arm doing the task. During an evoked brain activity, regional changes in blood flow alter the concentration of O2Hb in the brain (Clancy et al., 2014). Research has shown in healthy subjects, neural stimulation in the form of cognitive/motor tasks, can induce an increase in cerebral blood flow and O2Hb concentration, in which the increase in delivery of oxygen exceeds oxygen consumption as cerebral vasculature responds to physiological signaling messengers, i.e., calcium ions, nitric oxide, other metabolites (Davis et al., 1998). Significantly diminished brain oxygenation patterns have been observed in frontal and motor regions of the brain for post-concussion athletes 45 days post (Kontos et al., 2014), and TBI patients within hours after diagnosis (Roldan and Kyriacou, 2021), when performing motor and cognitive tasks. These studies hypothesized an altered cognitive resource allocation and compromised signaling messengers post-injury. However, our trends report increases in brain oxygenation changes which could be considered an immediate response, or specific to sub-concussive level impacts. Other studies that report increases in brain oxygenation for TBI patients when performing motor and cognitive tasks suggest the brain needs to work extra for similar tasks leading to overcompensation and increased brain oxygenation readouts (Rodriguez Merzagora et al., 2014). According to previous work, rotational velocities and accelerations even at lower levels, have been associated with diffuse brain tissue strains, which could lead to the disruption of neural connections especially at the repetitive level (Hernandez and Camarillo, 2019). This explanation would agree with the trends we observe in this study. It may also be that the type of cognitive load affects the brain oxygenation changes seen. One group reported that at lower cognitive load, a concussed group showed increased brain activity compared to controls, but in more difficult tasks the concussed group had diminished brain oxygenation compared to the control group (McAllister et al., 2001). This is similar to our results since in some cases there is an increase in brain oxygenation which may be greater during the ST period than the DT.

The goal of calculating sample entropy of fNIRS data was to include an additional measure to observe the regularity of the fNIRS series based on the existence of patterns in the data which could correspond to greater variations in concentration of hemoglobin. Entropy has been used as a mesure to detect cognitive impairments within dual-task functions in previous studies (Lamoth et al., 2011; IJmker, 2012). This is done to monitor possible hidden dynamical structures of motor function alterations due to dual-task abilities and identify potential cognitive impairment using the fNIRS signal (Ehsani et al., 2020). It is interesting to see greater sample entropy during the task sections of the fNIRS signal following the headers even though the athlete was performing the same task. This suggests that there was an increase in attentional resources and an elevated level of uncertainty in the motor function response as the signal became more unpredictable following the impacts. With more work, this additional analysis could have the potential to aid the fNIRS measurement as a biomarker for greater level head impacts in the future. Lastly, DT cost analysis of the fNIRS signal was included to account for musculoskeletal deficits following impacts. The average decrease may suggest that the skill level be increasing because subjects do not need the same attentional resources to perform the task, but this would need further testing since we do not see significant differences. Although, the level of sub-concussive soccer heading may not be enough to detect these changes in DT cost, this parameter may still be useful for higher level impacts in future studies.

We provide measurements from impacts for athletes performing 10 soccer headers from a ball launcher which is comparable to on-field scenarios of soccer headers in games or practice according to previous work (Kenny et al., 2022). On average, the female group experienced greater values of head kinematics including linear acceleration and angular velocity, which has also been reported in other studies, as well as higher rates of concussion among this group (Marar et al., 2012). In the current study, players were directed to stand still and return the ball to the same direction. Future studies could include allowing the players to freely move and direct the ball, replicating additional on-field scenarios. It has also been suggested that increasing neck muscle strength contributes to preventing large linear and angular accelerations while performing soccer headers (Caccese et al., 2018), which could also explain differences seen in male and female sports populations. We did not include this as a confounding factor in the current work, but this would be important for future studies.

The TCD portable imaging device allowed us to observe immediate individual changes of MCA blood flow velocities when comparing pre- and post-header measurements, but this trend did not hold across all participants. However, the female group differed from the male group with greater peak blood flow velocity after headers. The female group also experienced greater head kinematic values which may explain these differences. A previous study recorded posterior cerebral artery (PCA) and MCA blood flow velocity during a visual task (neurovascular coupling or NVC) using TCD, for seven male soccer athletes after performing 40 soccer headers, but did not observe changes in peak MCA or PCA velocities as well (Smirl et al., 2020). Interestingly, resting PCA and MCA velocity TCD measures have also been reported to remain unaltered days following concussion-level injury (Wright et al., 2017). Sample entropy analysis of the TCD signal was also different in these groups, before the headers, and almost significant following the headers. This could describe a change in the velocity profile or more complexity in the systolic and diastolic patterns for the different groups. There were also significant differences between male and female for the PI pre-header measurements. PI is known as a measure of flow resistance in the arteries and can be influenced by cerebral perfusion pressure, cerebrovascular resistance, arterial bed compliance, or HR (Thibeault et al., 2019). Increased PI has been reported in the case of severe TBI and led to increased intracranial pressure (O’Brien et al., 2015). Although we see significant differences in this parameter between males and females, this does not change after the 10 headers for either group. Overall, these results suggest that the TCD may not be sensitive enough to detect hemodynamic changes at this level of head impacts.

The UEF motor function test involves performing a novel and unfamiliar movement, which may require more motor cortex oxygenation changes (Kawai et al., 2015). Counting is considered a rhythmic task that involves working memory and is more directly related to executive function (Montero-Odasso et al., 2009). Combining these rhythmic tasks of different frequency may cause interference or be difficult to execute since DT recruits the same resources at the same time (Beauchet et al., 2005). The combination of skill-learning and counting backward creates a stress test in which the brain may reveal changes in terms of impairment. However, there was no change in the cognitive score calculated from the arm sensor movements nor balance parameters, suggesting no immediate change after 10 sub-concussive impacts. Studies have suggested neuropsychological function determined by neurocognitive tests is unaffected by repetitive sub-concussive level head impacts (Belanger et al., 2016). Previous work has shown that balance performance of healthy athletes returns to baseline levels within 20 min of rest following 30 min of physical activity (Susco et al., 2004). In our study, balance is the last parameter measured following TCD and fNIRS, which is measured near that 20-min mark and could explain why there are no significant changes observed. These aspects were still investigated because soccer heading has previously been associated with neurocognitive deficits and changes in balance long term (Svaldi et al., 2017).

Lastly, the HR measured while athletes performed the DT did not show significant differences to HR when performing soccer heading. Participants remained relatively still and only made head contact with the ball during the 10 headers with minimal exercise. During exercise, the heart beats faster and healthy blood vessels will expand in size to allow increased blood flow and help maintain blood pressure. No change in HR from the arm motor movement to heading may suggest that hemodynamic changes observed in this study are largely due to the impact to the head and not the autonomic nervous system response or changes in HR. In addition, there were no correlations of fNIRS concentration values with change in HR for any brain regions following the headers, and the control group did not exhibit any significant changes in HR or O2Hb concentration, which further supports this assumption.

### 4.1. Limitations

There are several limitations to this study. The smaller sample size of soccer athletes from one institution may have contributed to a reduced number of significant findings. Future work should include larger sample sizes with varying levels of soccer athletes, as well as a control group that would undergo similar movement without contact to the head. Additionally, the bite-bar sensor has limitations in its ability to measure head impacts more accurately. With the sensor being placed at the end of the bar and outside the mouth, there is chance for cantilever resonance that could affect the recordings (Camarillo et al., 2008). For this reason, the players were instructed to clench onto the bite-bar as tightly as possible during the headers. The UEF DT test as well as balance measures may also be limited to a learning curve from the participants with the pre- and post-header measurement design. To minimize this, a trial period was conducted before any measurements took place. Additionally, we did not include HHb concentration change results in this study and would need further investigation as not many patterns were found in our data. A few studies have demonstrated that oxyhemoglobin is a more sensitive marker of task-related brain oxygenation compared to deoxyhemoglobin (Wolf et al., 2002; Mihara et al., 2007; Harada et al., 2009), which is why we only include this parameter in our results.

## 5. Conclusion

This work has provided multiple measures to show that changes in brain physiology can be observed hyper-acutely resulting from sub-concussive impacts using portable, non-invasive, real-time imaging techniques. Our findings report interesting changes in fNIRS imaging in terms of increased concentration changes post-impacts. Further analysis of fNIRS signals such as sample entropy has shown significance in post-header measurement changes as well, which could serve as a possible biomarker for hyper-acute changes from impacts and applicable in future work. Immediate on-field measurements on repetitive head impact exposure and hemodynamic response could lead to improved monitoring and management of real-world head impacts in sports and making decisions to best minimize the magnitude and number of reoccurring head impacts during sport participation.

## Data availability statement

The raw data supporting the conclusions of this article will be made available by the authors, without undue reservation.

## Ethics statement

The studies involving humans were approved by Human Subjects Institutional Review Board at the University of Arizona. The studies were conducted in accordance with the local legislation and institutional requirements. The participants provided their written informed consent to participate in this study. Written informed consent was obtained from the individual(s) for the publication of any potentially identifiable images or data included in this article.

## Author contributions

CG is the primary author and contributed to the design of the research questions, the protocol conducted, data collection, data analysis and interpretation, and drafting and revising of the work. DH contributed to the data collection and data analysis for the entire study. LW contributed substantially to the revision of the drafted work, and provided the custom IMU methods for the tri-axial bite-bar sensor. NT contributed to the interpretation of data as well as substantial revision of the drafted work. KL contributed to the design of the study, data interpretation, and substantial revising of the drafted work. All authors listed have made a substantial contribution to this work in terms of design, data acquisition, data analysis, or interpretation of data.

## Conflict of interest

The authors declare that the research was conducted in the absence of any commercial or financial relationships that could be construed as a potential conflict of interest.

## Publisher’s note

All claims expressed in this article are solely those of the authors and do not necessarily represent those of their affiliated organizations, or those of the publisher, the editors and the reviewers. Any product that may be evaluated in this article, or claim that may be made by its manufacturer, is not guaranteed or endorsed by the publisher.
