# Supplementary material for: Hyper-acute effects of sub-concussive soccer headers on brain function and hemodynamics
Source: Front Hum Neurosci. 2023 Sep 14;17:1191284. doi: 10.3389/fnhum.2023.1191284 (PMC10538631; doi:10.3389/fnhum.2023.1191284)
Supplement: Supplementary file 1 [file Data_Sheet_1.docx]

Supplementary Material

Hyper-acute effects of sub-concussive soccer headers on brain function and hemodynamics

Carissa Grijalva^1^, Dallin Hale^2^, Lyndia Wu^3^, Nima Toosizadeh^1,4^, Kaveh Laksari^1,5*^

*** Correspondence:** Kaveh Laksari: klaksari@arizona.edu

# Supplementary Figures and Tables


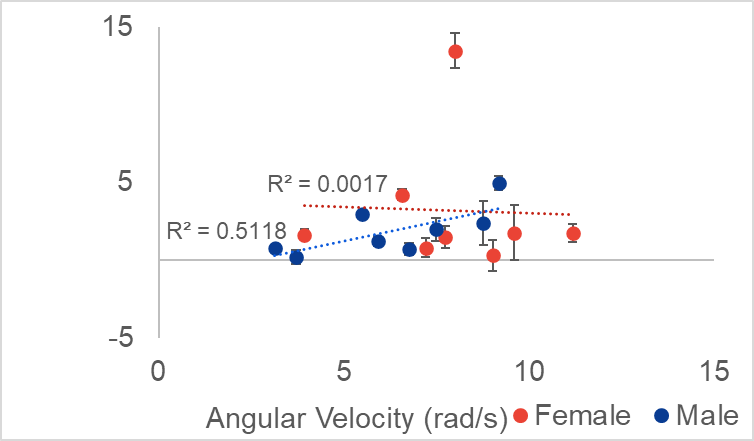

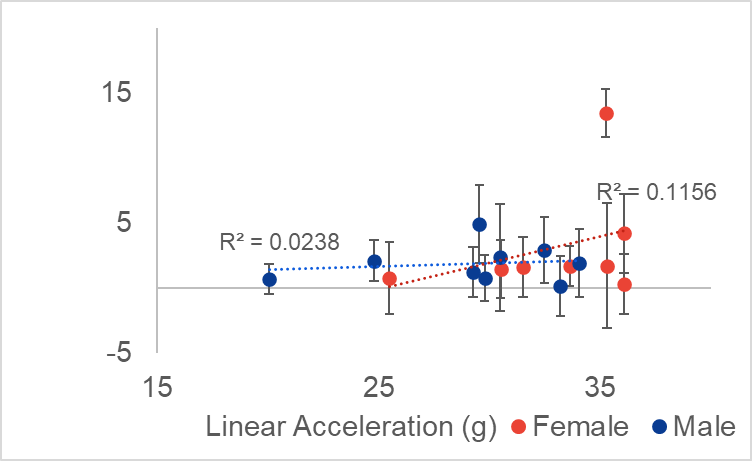


**Supplementary Figure 1.** Individual difference in blood flow velocity vs acceleration or angular velocity. Error bars represent standard error.


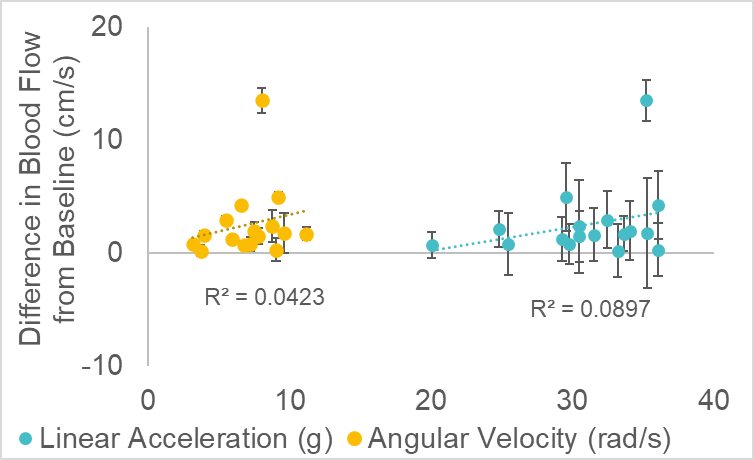


**Supplemental Figure 3.** Left: Heart rate during pre- and post-header measurements, and during soccer headers. Error bars represent the standard error. Right: p values for correlation of heart rate range and fNIRS measurements.


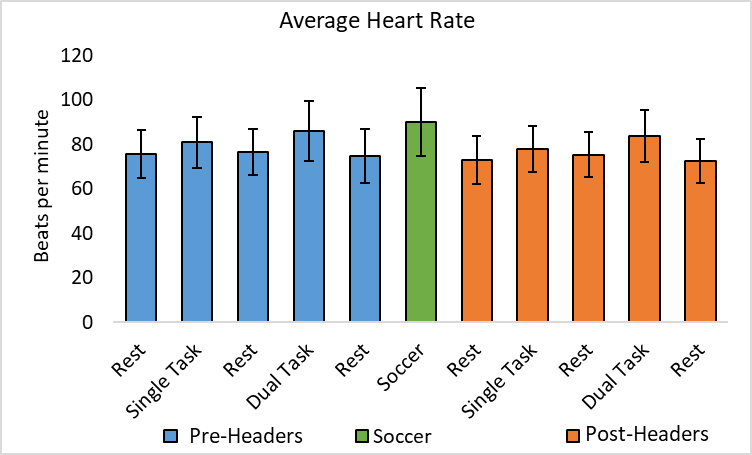


**Supplementary Figure 2.** Peak value O2Hb concentration changes for the (A) Right Prefrontal cortex and the (B) Motor Cortex during the Upper Extremity Function Task ; Sample entropy pre- and post- measurements in the (C) left prefrontal cortex and the (D) motor cortex for oxyhemoglobin concentration. Error bars represent the standard error.


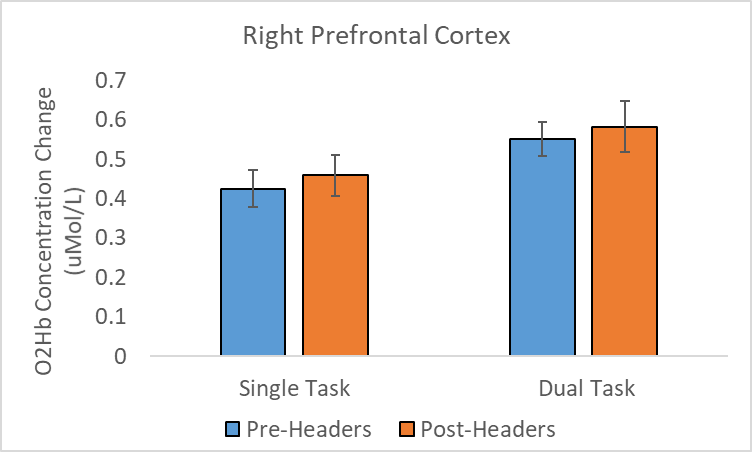

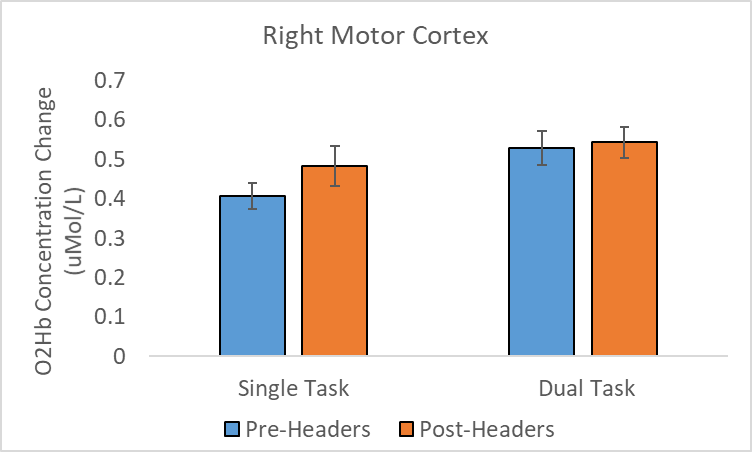

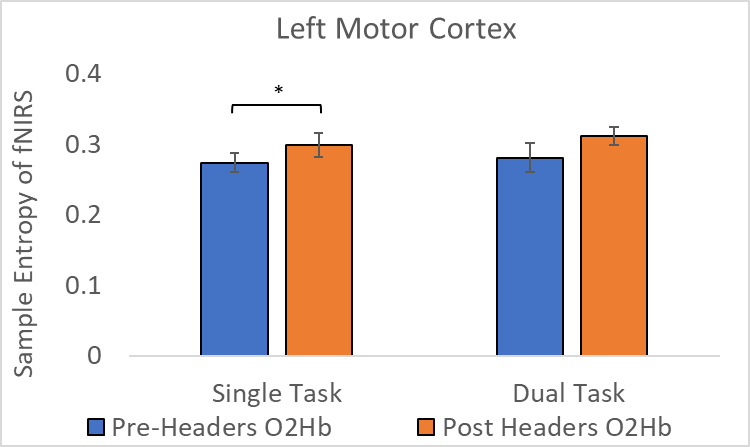

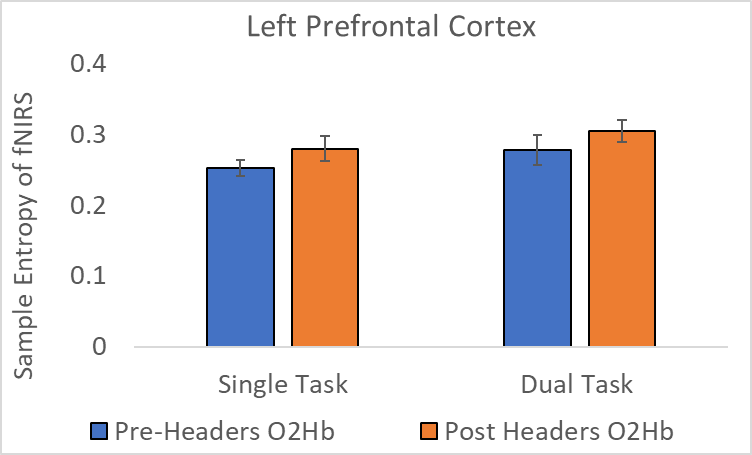


**(B)**

**(C)**

**(D)**

**(A)**

| Heart Rate Range (bpm) | fNIRS concentration (uMol/L) | P value |
| --- | --- | --- |
|  | Left Prefrontal |  |
| ST: 27.4±7.3 | ST: 0.51±0.2 | 0.09 |
| DT: 31.6±9.6 | DT: 0.62±0.3 | 0.34 |
|  | Right Prefrontal |  |
|  | ST: 0.45±0.2 | 0.4 |
|  | DT: 0.58±0.3 | 0.37 |
|  | Left Motor |  |
|  | ST: 0.55±0.3 | 0.13 |
|  | DT: 0.71±0.3 | 0.2 |
|  | Right Motor |  |
|  | ST: 0.48±0.2 | 0.07 |
|  | DT: 0.54±0.2 | 0.31 |


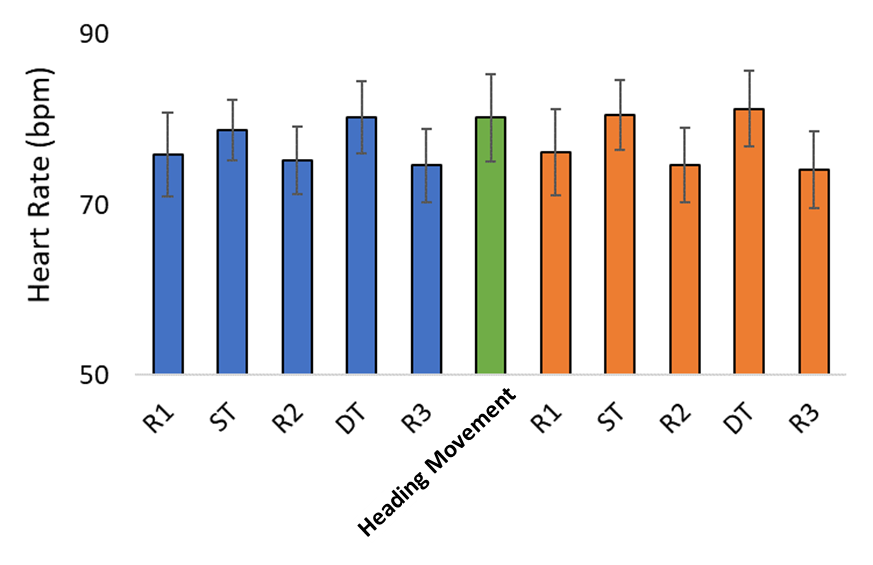


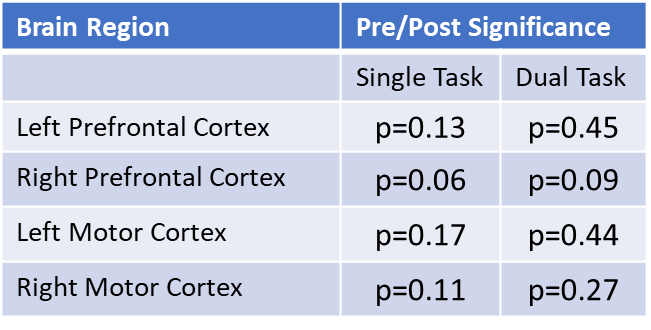


**Supplemental Figure 5.** Peak value HHb concentration changes for the (A) Left Prefrontal cortex and the (B) Left Motor Cortex during the Upper Extremity Function Task


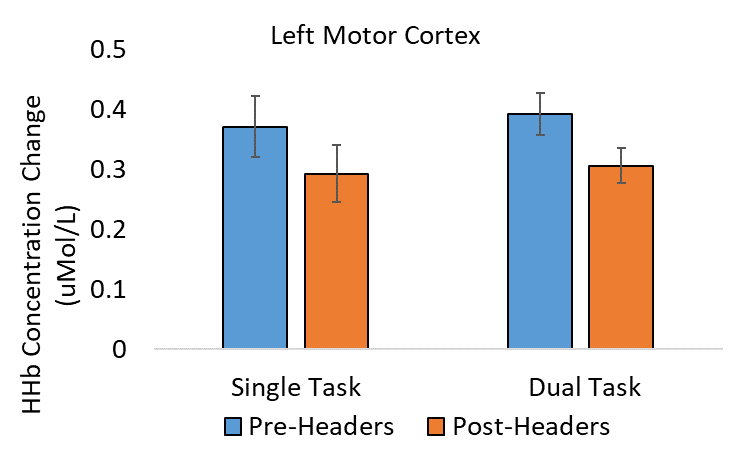

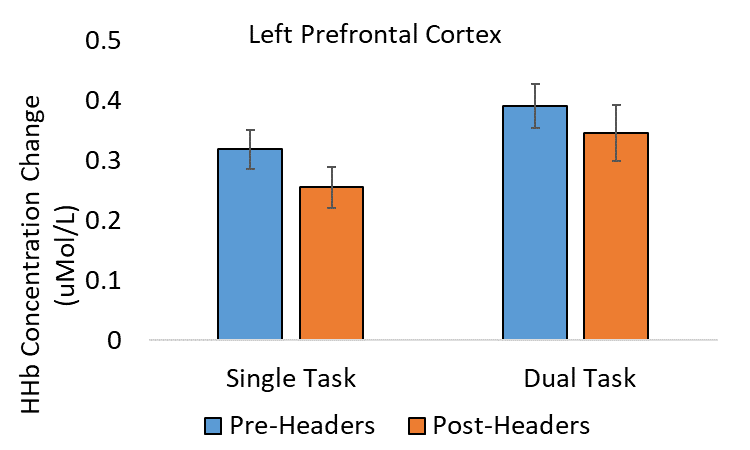


**Supplemental Figure 4.** Left: Heart rate during pre- and post-header measurements, and during soccer headers for control group. Error bars represent the standard error. Right: p values for pre- and post-measurement of the fNIRS O2Hb concentration values.


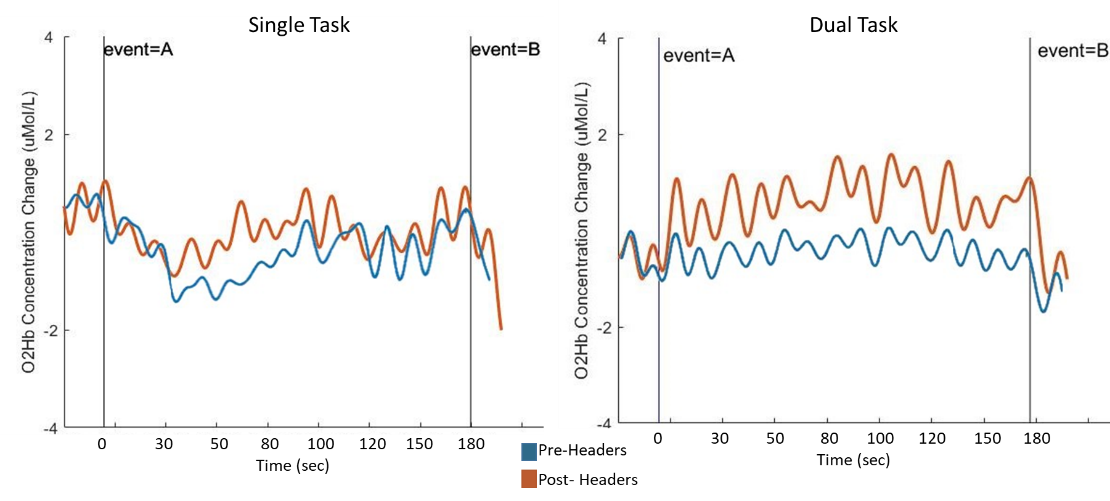


**Supplemental Figure 6.** Sample of pre- and post-header values for O2Hb concentration change during the fNIRS single task and dual task tests. Event A signals the start of the task, and Event B signals the end of the task.
